# Ultrasound-guided decompression surgery of the tarsal tunnel: a novel technique for the proximal tarsal tunnel syndrome—Part II

**DOI:** 10.1007/s00276-018-2127-9

**Published:** 2018-10-31

**Authors:** Alejandro Fernández-Gibello, Simone Moroni, Gabriel Camuñas, Rubén Montes, Marit Zwierzina, Christoph Tasch, Vasco Starke, José Sañudo, Teresa Vazquez, Marko Konschake

**Affiliations:** 1 Faculty of Health Sciences, Department of Podiatry, University of La Salle, Clinic Vitruvio Biomecánica, Madrid, Spain; 2 Faculty of Health Sciences at Manresa, Department of Podiatry, Universitat de Vic-Universitat Central de Catalunya (UVic-Ucc), Clinic Vitruvio Biomecánica, Barcelona, Madrid, Spain; 3 Department of Plastic, Reconstructive and Aesthetic Surgery, Center of Operative Medicine, Medical University of Innsbruck, Innsbruck, Austria; 4 Department of Anatomy, Histology and Embryology, Division of Clinical and Functional Anatomy, Medical University of Innsbruck (MUI), Müllerstr. 59, 6020 Innsbruck, Austria; 5 Anatomy and Embryology Department, School of Medicine, Complutense University of Madrid, Madrid, Spain

**Keywords:** Tarsal tunnel syndrome, Heel pain syndrome, Ultrasound, Minimally invasive foot and ankle surgery, Ultrasound-guided, Nerve entrapment

## Abstract

**Background:**

The aim of this study is to provide a safe ultrasound-guided minimally invasive surgical approach for a proximal tarsal tunnel release concerning nerve entrapments.

**Methods and results:**

The study was carried out on ten fresh-frozen feet. All of them were examined by high resolution ultrasound at the medial ankle region. The surgical approach was marked throughout the course of the flexor retinaculum (laciniate ligament). Once the previous steps were done, the flexor retinaculum release technique was carried out with a 2-mm entry only. As a result, an effective and safe release of the flexor retinaculum was obtained in all fresh-frozen feet.

**Conclusion:**

The results of our anatomic study indicate that our novel ultrasound-guided minimally invasive surgical approach for the release of the flexor retinaculum might be an effective, safe and quick decompression technique treating selected patients with a proximal tarsal tunnel syndrome.

## Introduction

The tibial nerve (TN) often—mistakenly—in current literature also named the “posterior tibial nerve”, takes its course within osteofibrous tubes and is vulnerable to an entrapment. The entrapment of the TN within the tarsal tunnel (TT), also known as the tibio-calcaneal tunnel, calcaneal tunnel or Richet´s tunnel can occur in two different osteofibrous tubes: in the proximal tarsal tube and the distal tarsal tube [10].

The tarsal tunnel syndrome itself was first described by Keck et al. and by Lam et al. in two independent publications [22, 30]. Its signs and symptoms were already examined in 1960 by Kopell and Thomson et al. [27]. The true incidence is unknown; a specific cause of the tarsal tunnel syndrome (TTS) can only be identified in 60–80% of the patients [24, 31] and has a slight female predominance (56%) [7]. 50% of the cases are idiopathic with no identifiable cause [13].

Plantar heel pain has a prevalence of 11–15% in adults with foot problems and it has been seen that up to 88% of the patients with chronic heel pain have some degree of nerve compression [2]. Cimino et al., in their review over 186 feet, could show that the most common causes of tarsal tunnel syndrome were as follow (in order of incidence): idiopathic, traumatic, varicosities, heel varus, fibrosis, heel valgus, ganglion, diabetes, obesity, tight tarsal canal, hypertrophic abductor hallucis muscle, rheumatoid arthritis, lipoma, anomalous artery, acromegaly, ankylosing spondylitis, regional migratory osteoporosis and the flexor digitorum longus muscle [7]. According to Sammarco et al., the most common findings in 72 patients with positive electrodiagnostic studies were: scar, vascular leashes and varicosities, accessory flexor digitorum longus muscle, tight fascias, bony exostosis, neuroma, the retinaculum and ganglions [2]. For Takakura et al., the most common causes were ganglion, tarsal coalition, idiopathic, trauma and tumors [50].

When an idiopathic diagnosis for a TTS has been made, many authors have attributed the etiology to a fibrosis and thickening of the flexor retinaculum (FR), leading to an increase of the tube pressure [12, 14, 32, 39, 47]. This fascial structure, also called medial annular ligament or lacinate ligament is formed by two layers, a superficial and a deep layer, with a proximal anteriorly oriented apex and an inferior base along the superior border of the abductor hallucis muscle.

The deep sling of the FR inserts on the anterior aspect of the medial malleolus, after running deep to the tibialis anterior tendon and inserts on the deep sling of the superior extensor retinaculum. The posterior border of the FR extends from the tip of the medial malleolus to the posterior–superior aspect of the calcaneus medially. The deeper layer covers medially the tibialis posterior tendon (TP), flexor digitorum longus (FDL) and flexor hallucis longus (FHL) muscle and inserts in the upper medial surface of the posterior tuberosity of the calcaneus, while the superficial one envelope the lower third of the calcaneal tendon medially. Between those two layers of the FR lies the tibial neurovascular bundle [23].

The normal pressure of the tarsal tunnel, according to Trepman et al., is about 2 ± 1 mmHg in mild plantar flexion and neutral eversion/inversion. The pressure increased to 32 ± 5 mmHg in full eversion and 17 ± 5 mmHg in full inversion [53]. Rosson et al. validated the hypothesis that patients who received a diagnosis of a tarsal tunnel syndrome had higher tunnel pressures; with these structures released, the pressure immediately decreased [44]. For decompression of the proximal tarsal tunnel, there exist many surgical procedures since the very first description of the tarsal tunnel syndrome. All of those techniques were described as an open surgical procedure, which demonstrated functional improvement in those patients with symptoms severe enough to require surgery [45]. Nevertheless, there have been described complications after open procedures that could result in wound dehiscence, infections and, most important, fibrotization of the tissues. More recently, it has been described a minimally invasive proximal tarsal tunnel release [9, 15] performed in a selective cluster of patients which has demonstrated reductions in complications compared to open procedures; until now, there is no ultrasound-guided, ultra-minimally invasive sonosurgery technique described. Our hypothesis, to release the superficial layers of the FR, between the FDL tendon and the neurovascular bundle within the 4 cm of this fascial structure [12], could decompress the neurovascular bundle with improvements in a selected cluster of patients, which suffer of a TTS.

Thus, the aim of this study is to prove the effectiveness and safety of an ultrasound-guided ankle and foot decompression surgery technique for a proximal tarsal tunnel release (UGAFDS).

## Materials and methods

The individuals had given their written informed consent prior to death for their use for scientific and educational purposes. According to National Law, scientific institutions (in general Institutes, Departments or Divisions of Medical Universities) are entitled to receive the body after death mainly by means of a specific legacy, which is a special form of last will and testament. No bequests are accepted without the donor having registered their legacy and been given appropriate information upon which to make a decision based upon written informed consent (policy of ethics); therefore an ethics committee approval is not necessary [26, 34].

For this “in vitro” study, we performed an US-guided surgical approach on 10 fresh-frozen feet (6 male, 4 female), which belong to the Body Donation Centre of the Complutense University of Madrid, Spain. We dissected the medial ankle region to evaluate the effectiveness and safety of the technique. All important structures could be protected; the FR could be released completely.

The exclusion criteria for this study were: BMI above 30 (impaired US echogenicity), sings of trauma of the ankle region, a history of ankle or foot ischemic vascular disorders, surgery or space mass occupancy injuries.

### Foot position, ultrasonographic anatomy and “STEP-BY-STEP” approach

All feet examined were positioned in decubitus supine position (the so-called “frog-leg position”), with a slight dorsiflexion of the ankle.

The normal US-imaging of the FR was obtained in an US long axis at the DM-line, appearing as a hyperechoic fibrillary structure with less than 1 mm of thickening in normal patients [54].

**Fig. 1:**
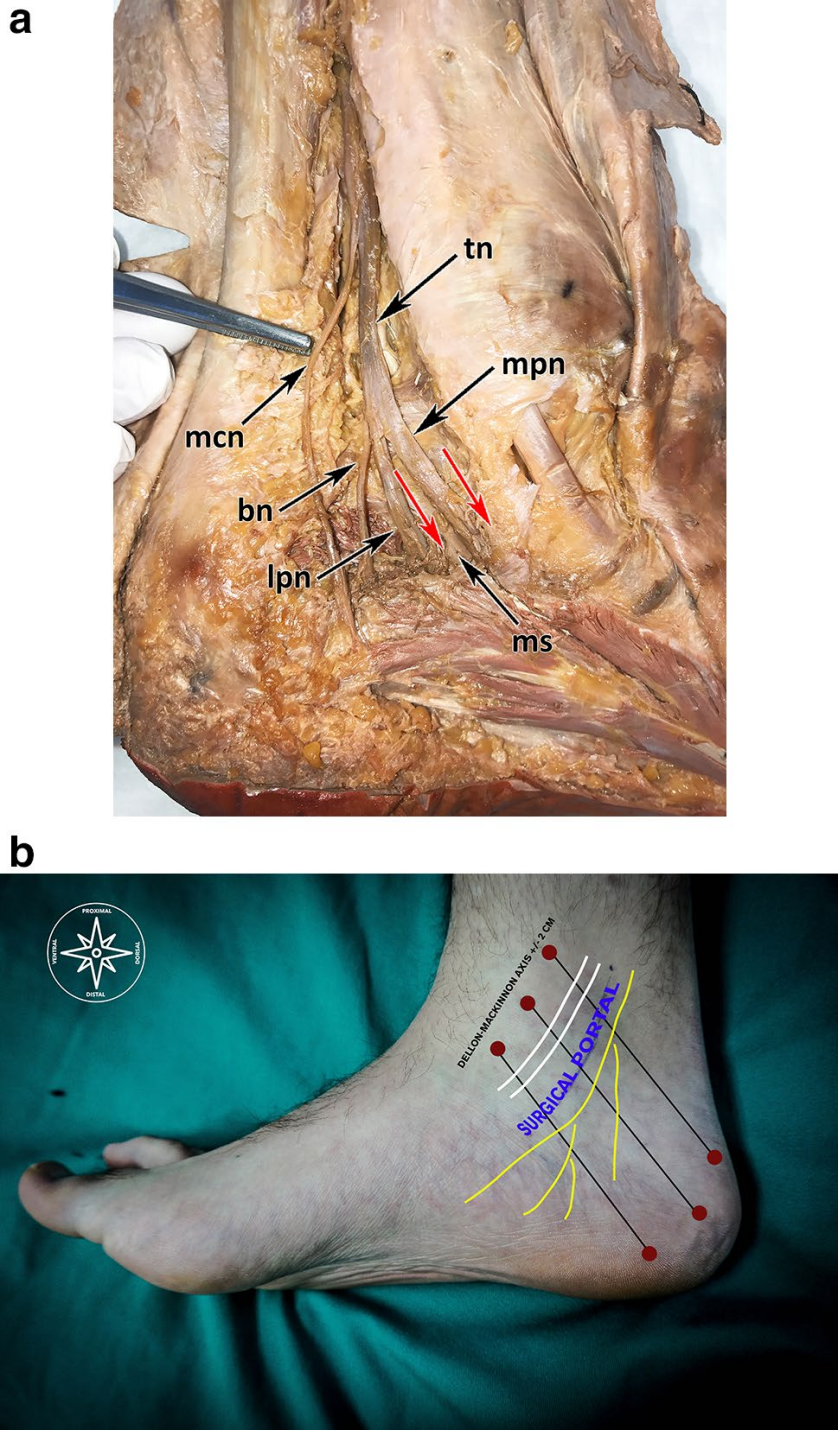
**a** Anatomical dissection of the tibial nerve and its branches running in the TT. *tn* Tibial nerve, *mpn* medial plantar nerve, *lpn* lateral plantar nerve, *bn* Baxter nerve, *mcb* medial calcaneal branch, *ms* medial intermuscular septum, red arrows: nerves entering separated tubes. **b** The red circles show the landmarks to sketch the flexor retinaculum; the black lines show the DM-line and the lines ± 2 cm (proximal and distal) to the DM-line; the yellow sketched lines show course of the tibial nerve with its branches; the white lines show the courses of the posterior tibial and the flexor digitorum muscles. (Color figure online)

An anatomic overview of the medial ankle region including the important landmarks is shown in Fig. 1a, b.

**Fig. 2:**
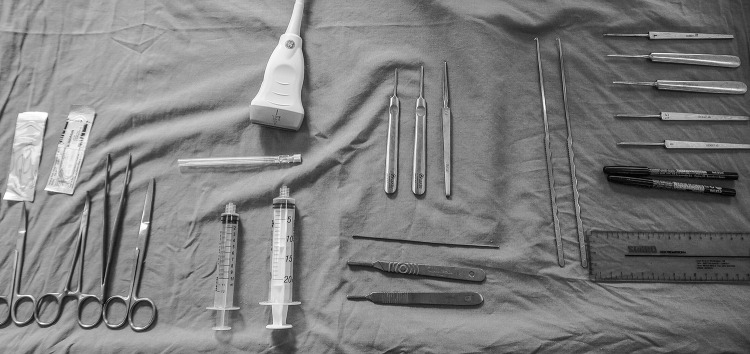
Instruments for the UGAFDS approach; ultrasound device; dissection material; 20 gauge needles, syringe; V-shape; hook knife

The “Step-by-Step” approach of the ultrasound-guided decompression surgery for the proximal tarsal tunnel syndrome was carried out as follows:

*Step I* The equipment used performing the UGAFDS for the proximal tarsal tunnel release were as follows (Fig. 2): High frequency ultrasound with 13 MHz (General Electric Logic R7), 20-gauge needle and a syringe of 10 cc, V-shape gouges of 1, 2, 3 mm, a hooked knife and a buttoned probe.

**Fig. 3:**
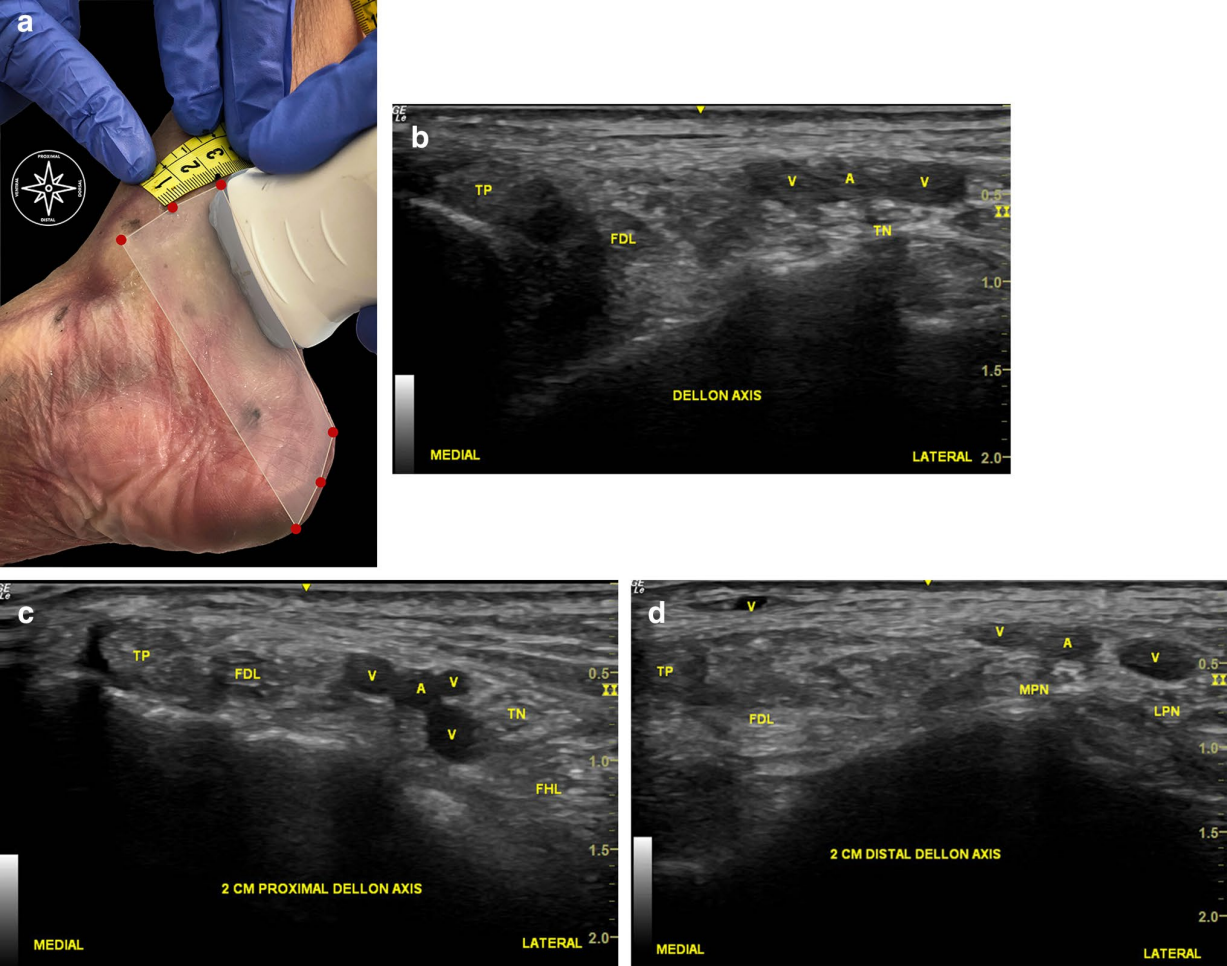
**a**–**d** The US pictures were taken 2 cm proximal and distal of the DM-line and directly at the level of the DM-line; the red circles are landmarks visualizing the flexor retinaculum; The yellow head arrow mark the surgical portal at the US pictures; *TP* tibial posterior, *FDL* flexor digitorum longus; *V* posterior tibial veins and perforating vein at the 2 cm distal Dellon line, *A* posterior tibial artery, *TN* tibial nerve, *MPN* medial plantar nerve, *LPN* lateral plantar nerve, *FHL* flexor hallucis longus muscle. (Color figure online)

*Step II* Before performing the surgical technique, we defined the landmarks and performed the US measures (Fig. 3a–d): At first, we drew the DM-line and the lines 2 cm proximal and distal to the DM-line and examined the surgical portal with the US. Subsequently, we marked the points where the branches of the TN crossed those three lines, putting the US probe over the three lines, examining the nerves in the short axis and drew a line through these points marking the course of the nerves on the skin.

**Fig. 4:**
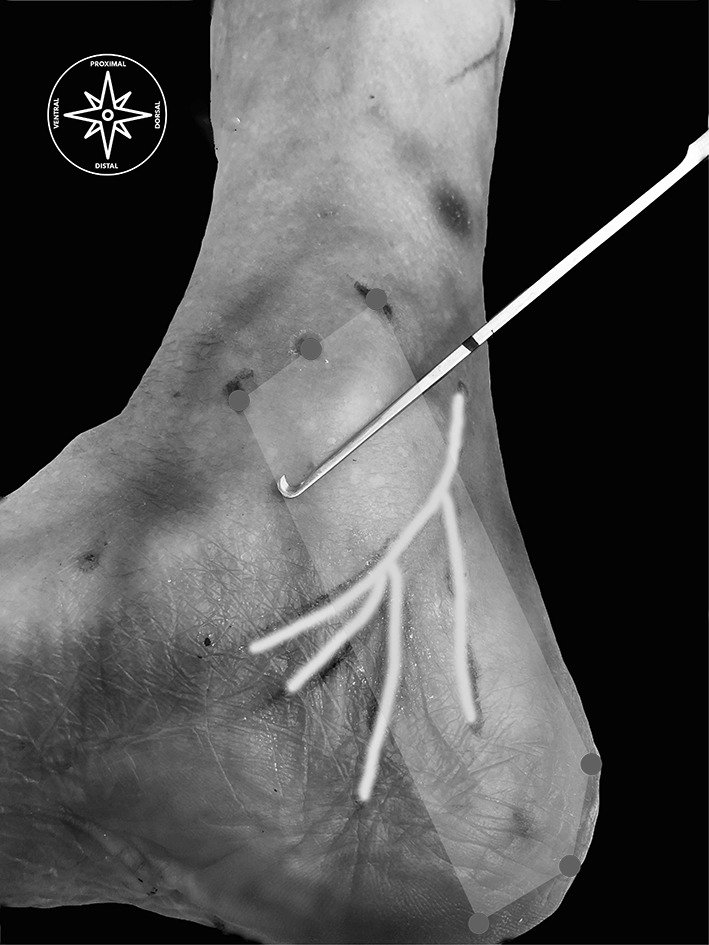
Red circles show the landmarks to sketch the flexor retinaculum; the yellow lines represent the tibial nerve and its branches; the instrument is the hooked knife over the surgical portal. (Color figure online)

With the same US procedure, we marked a point for each of those lines, drew a line through these points so that one can visualize on the skin the course of the instruments for a safe surgical US-guided procedure (Fig. 4).

*Step III* The next step consists of introducing a 20-gauge needle previously curved at 90° at the proximal part from the proximal point of our “surgical line” previously drawn. With the 20-g needle we performed the US-guided hydrodissection, at the same time performing US pictures at the three DM lines to make sure that we could increase the surgical approach.

*Step IV* Using the needle as a guide, under continued US-guidance we introduced the V-shape gouge of 1 mm, followed by the two V-shape of 2 mm gouge from the same portal to enlarge the “working space” to insert the hooked knife.

*Step V* We created a safe channel that allowed us to introduce a 3-mm hooked knife with the blade rotated towards the flexor digitorum longus, to minimize every possible dangerous contact with the nerve-vessel bundle. Once we had the tip of the hooked knife at the 2 cm distal line from the DM-line, we rotated the hook towards the flexor retinaculum, performing the retrograde cut under US guidance.

**Fig. 5:**
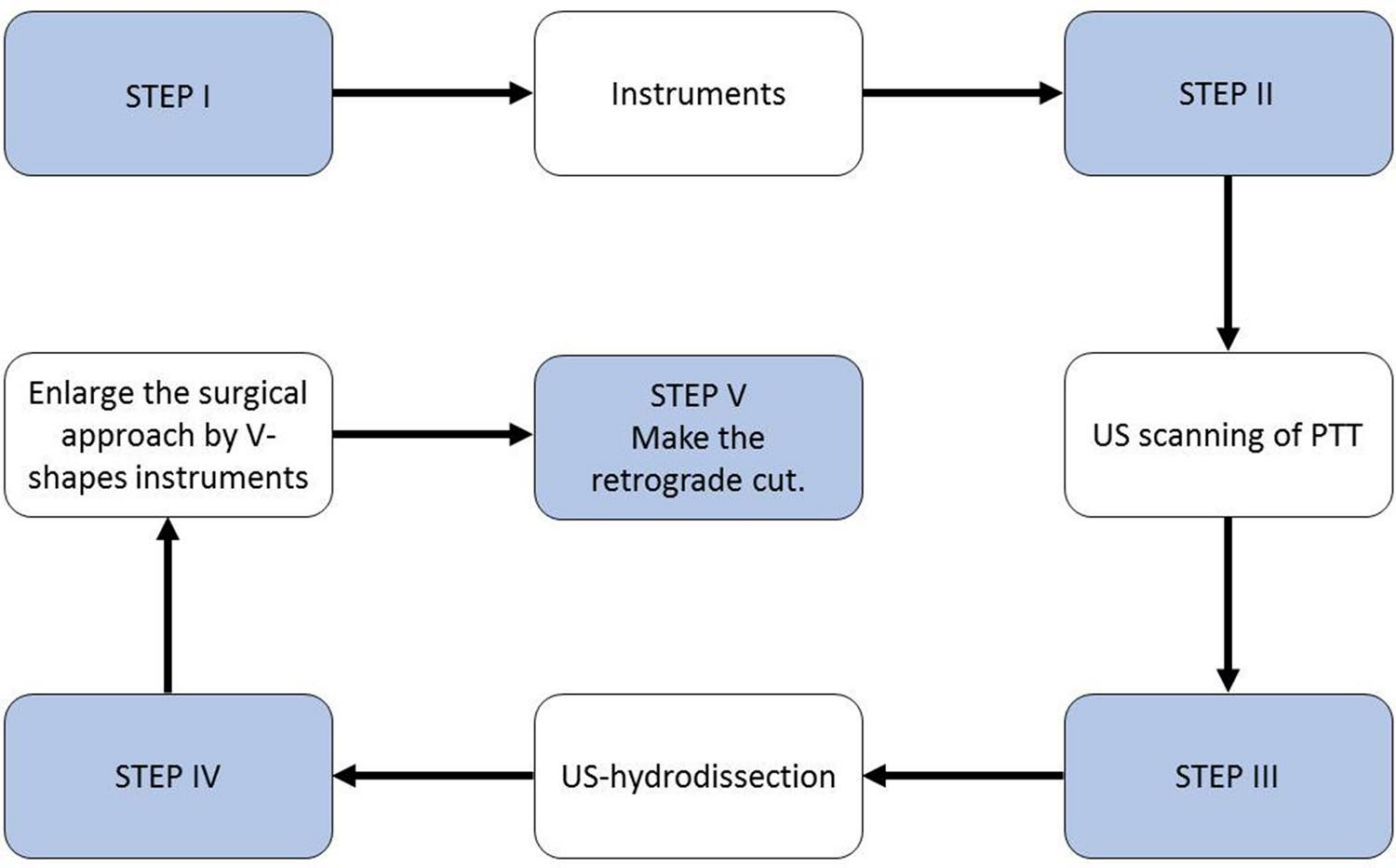
The new surgical approach is summarized in five easy steps: the STEP I: take the material; STEP II: perform a US scanning of the tarsal tunnel; STEP III: make the US-hydrodissection with the 20 gauge needle and the syringe; STEP IV: using the needle as a guide, we introduce the V-shapes in increasing order of size; STEP V: with the hook knife, we make the release of the flexor retinaculum

**Fig. 6:**
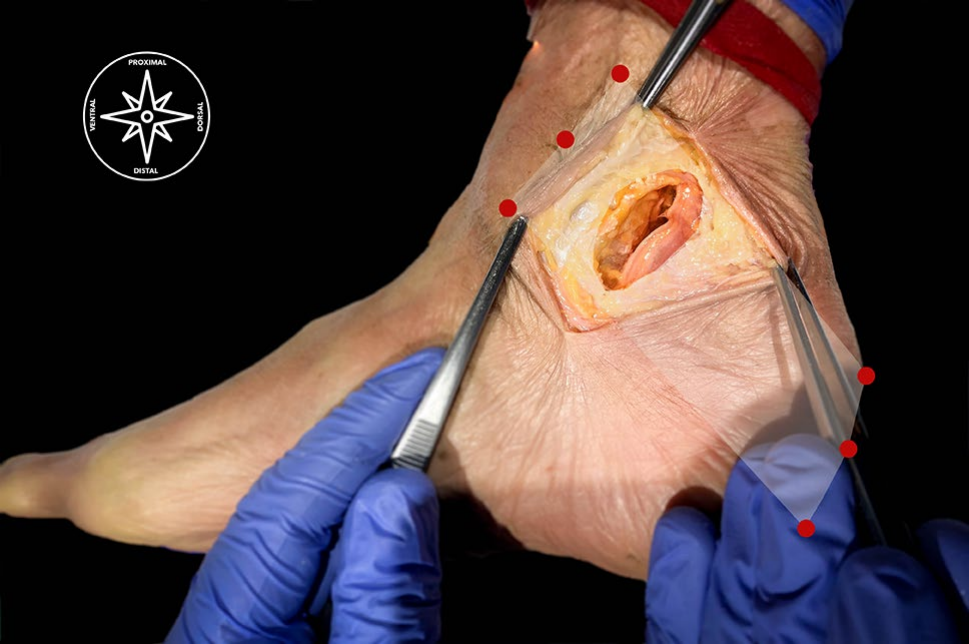
Red circles are the landmarks to sketch the flexor retinaculum; after the minimally approach and dissections one can see the result cutting the whole flexor retinaculum at the proximal tarsal tunnel. (Color figure online)

Figure 5 summarizes our surgical approach with five easy steps; Fig. 6 shows the final result.

After finishing the surgical approach, we performed an US proof to be sure the whole flexor retinaculum has been cut. Additionally, also under US guidance, we inserted a probe (buttoned probe) in the surgical canal pushing it towards the skin for another proof of a total decompression.

During the surgical approach, at the first step (landmarks—US measures), we scanned all the feet without finding any morphological alteration in the tunnel or any injury due to space occupation or anatomical alteration. When we check that everything is correct in the ultrasound scan, we drew the marks with a dermographic pencil. Within the next step (hydrodissection), with a 20-gauge needle, we inserted it proximal to the flexor retinaculum, in the short US axis, assuring us that we are in the surgical entry point (between the flexor digitorum longus and the nerve-vessel bundle). We inserted the saline, while we see how the bundle moved away and we made a wider and safer surgical entry point. In all feet examined, we could confirm that space increased and the bundle moved away.

**Fig. 7:**
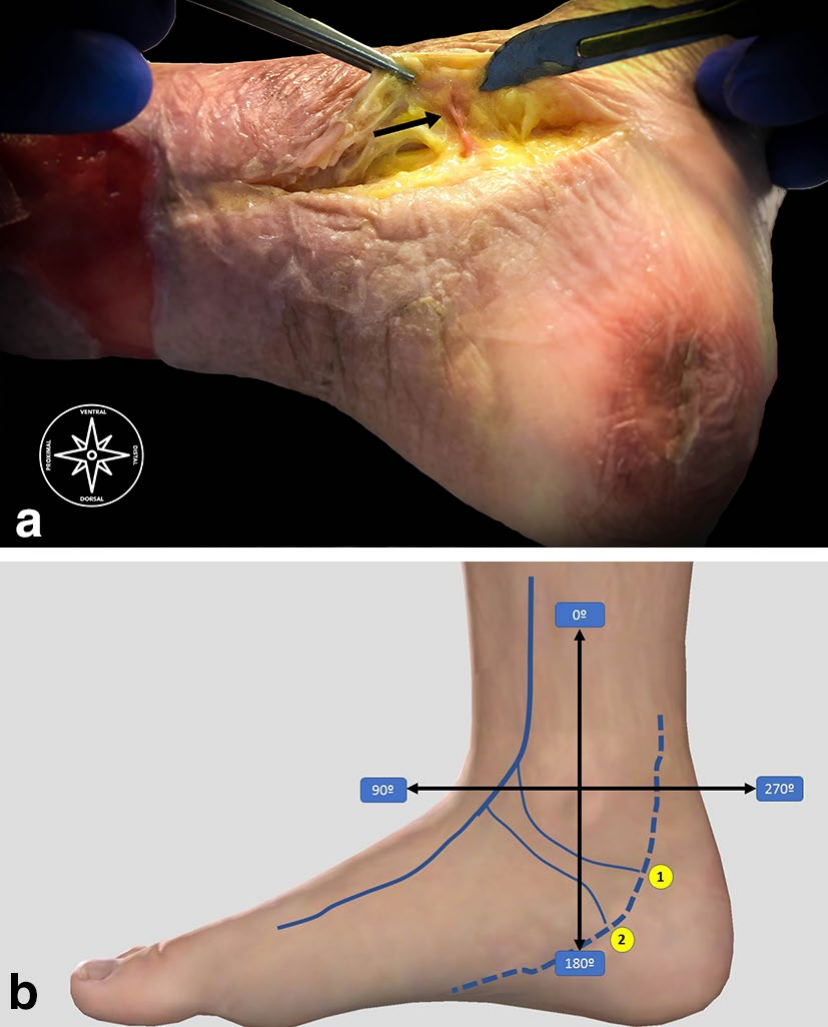
**a** Shows an anatomical dissection with the constant perforating vein; in **b** the sketch shows both a constant and an inconstant perforating vein and their location

The next step (the US-surgical procedure), without removing the needle from the hydrodissection, we inserted the V-shapes in increasing order, taking care to follow the guide, performing the perforations at the same point. We followed introducing the hooked knife 4 cm along the working space, with the cutting edge looking to the flexor digitorum longus muscle, finally turned it upward and made the retrograde cut preserving the perforating veins, skin and all important structures (Figs. 6, 7a, b**)**.

## Results

The median average duration of the procedure, including all the surgical approach, was approximately of 15 min ± 3 min, decreasing in time for each procedure due to the learning curve.

In all 10 feet examined the osseus landmarks were clearly identifiable, despite 4/10 feet got a BMI between 25 and 30.

In the pre-surgical scanning, we were able to identify the TN and all its branches in all ten subjects.

The median anatomical US measure of the FR of our 10 specimens was 0.83 mm.

The mean length of the incision was 2.5 mm (± 0.5 mm) within the cluster of 10 feet, so that we were able to categorize our approach as an “ultra-minimally invasive surgery”.

After dissection by a senior anatomist with more than 10 years of experience, we measured the cut at the FR with a measure of 4 cm ± 3 mm.

Our results on 10 feet (six male and four female) showed, that it is a safe technique to perform the release of the flexor retinaculum without damaging important structures. For reducing and minimizing possible complications it is important to always be under US-guidance during the cut and to cut the flexor retinaculum without damaging superficial veins, the subcutaneous tissue or the skin.

## Discussion

To get a better diagnosis and treatment of the tarsal tunnel syndrome, it is indispensable to know the normal tibial nerve anatomy and all branching patterns even of all small branches. Due to the complex topography of the TN branches within the tarsal tunnel in relation with all the osteofibrous structures, the presurgical US—scanning plays an important role of the management of this overlooked syndrome. For example, own investigations showed that the medial calcaneal nerve (MCN) in 40% of the cases branches off proximal to the tunnel and in other 25% two MCN can arise from the TN, one running within the tunnel, the other one not. Such relevant findings are of crucial importance for all surgical procedures in this area [12]. It could be shown that there is no safe zone at the medial aspect of the calcaneus for “blind” surgical techniques [21].

Ultrasound is an imaging technique, which is currently growing enormously in musculoskeletal (MSK) disorders. It is not only used as a diagnosis device, but also for MSK treatment, both for invasive and surgical [5, 40–42, 49]. Modern ultrasound devices are a very useful tool, which nowadays allow us to identify different and even small nerve branches with a great anatomic variability [19]. We already demonstrated in the first part of our study series, that ultrasound is a very useful tool, especially in the ankle and heel region. By defining an algorithm for ultrasonographic routine implementation, we presented an easy to follow method to locate all important topographic structures of the medial ankle region.

According to Nagaoka et al., as a diagnostic imaging technique for the tarsal tunnel syndrome, High Resolution Ultrasound (HRUS) is also useful for identifying space-occupying lesions [36]. It is highly recommended to perform routinely US in patients with a tarsal tunnel syndrome [36, 37].

Tawfik et al. postulated, that high resolution ultrasound is a good tool for the diagnosis of a tarsal tunnel syndrome [52]; they proposed using US to measure the “cross-sectional area” with good results in sensitivity and specificity; nevertheless there are more studies needed for confirmation [1]. Alshami demonstrated that the intra- and inter-provider reliability was excellent in measuring nerves through US with tiny measurement errors, and one has to keep in mind that a side-to-side difference as small as ∼ 1.8 mm^2^ has been seen to be meaningful in an individual patient. This difference is much smaller than the swelling reported in the literature for patients with tibial neuropathy [2].

The “Gold Standard” test to diagnose the carpal tunnel syndrome is the electromyography (EMG) although physicians need to evaluate any mass occupying space. In contrast, in the literature it has been estimated that the EMG only can identify the tarsal tunnel syndrome in 50–80% of the cases, in the remaining 30–50% of the cases it is diagnosed as idiopathic and the etiology is thought to be attributed to osteo-fibrous structure issues [13, 31, 38, 46].

Currently, in the most of the cases, clinicians are used to diagnose a TTS basing on symptoms, orthopedic tests and EMG [7]. The pain is usually characterized as burning, sharp, shooting, shock-like, electric, in nature localized or radiating either proximally or distally, worsening through the day, also occurring with rest and in non-weight bearing positions [4, 18].

Other sign is the post-static dyskinesia, especially with the very first steps in the morning such as for plantar fasciopathy, and that is why the diagnosis could be so misleading [20].

Paresthesia and neurological changes are other signs that Kinoshita et al. found mostly in MPN than in LPN [25]. Moreover, those signs are not common in the entrapment of the first branch of lateral plantar nerve (in literature also known as Baxter’s Nerve), because of its nature as a motor nerve [2].

Dorsiflexion–eversion test is another orthopedic relevant test for tarsal tunnel syndrome, according to Kinoshita et al. [25]. This test reproduced or aggravated the symptoms in the majority of patients (36 of 44 feet) because it has been seen that this position dramatically increases the tension at the tibial nerve [8]. Passive plantar flexion–inversion test may reproduce or aggravate symptoms increasing pressure on the tibial nerve at the tarsal tunnel [2]. Furthermore, there are many neurodynamic tests less known that can be performed as complementary tests for the diagnosis of a tarsal tunnel syndrome [6, 48].

Rose et al. and Tassler et al. in their studies highlight the importance of sensory testing, to investigate sensory dysfunctions in patients with plantar heel pain or tarsal tunnel syndrome [43, 51].

The Tinel test has been shown to be one of the clinical tests with great accuracy, eliciting pain when the nerves are submitted to acute or chronic compression [11, 17].

According to Watson et al. surgery is considered after 12 months of conservative treatment; the American Orthopaedic Foot and Ankle Society itself recommend a non-operative treatment for 12–18 months [55].

Some authors use the procedure described by Lam and Watson et al., in which both tunnels should have been released, the proximal and distal tarsal tunnel. In their study, it has been highlighted the importance of a decompression of the tarsal tunnel until 1 inch distally to the sole of the foot [30, 55].

Mann et al. recommend starting the incision 10 cm proximal to the distal tip of the medial malleolus and 2 cm dorsal to the posterior margin of the tibia [33]. Antoniadis et al. said that the surgical approach consists in decompression of the TN and its branches over a long segment, from the lower third of the calf proximally down to the distal medial portion at the sole of the foot [3]. Furthermore, Wieman et al. described an incision 5 cm above and 1 cm posterior to the medial malleolus until a point 1–2 cm below and 2 cm anterior to that [56].

For Gould et al. the incision has to begin proximal to the flexor retinaculum and continues up to three quarters of the sole of the foot transversally crossing [16].

A lot of described techniques focus on releasing the flexor retinaculum and the deep fascia of the abductor hallucis muscle and also, if needed, to perform a neurolysis of the tibial nerve and its branches [3, 28, 30, 35, 44, 56]. It also could be shown that the release of the proximal and distal tarsal tunnels improves nerve conduction and creates hemodynamic changes [24, 44].

In our study we described, for the first time, a minimally invasive, ultrasound-guided ankle and foot decompression surgery (UGAFDS) technique for the proximal tarsal tunnel compartment, which consists of the release of the flexor retinaculum.

As already previously discussed, there might be many etiological factors which can lead to a tarsal tunnel syndrome, but often it is not possible to make a diagnosis due to a macroscopic or well visible cause such as a mass occupying space or due to a metabolic disease such as diabetes [56]. Therefore, a release of the flexor retinaculum reducing the intracompartmental pressure has been described to be the main surgical goal [22]. Thus, future clinical studies are necessary to ascertain all the causes of a tarsal tunnel syndrome to provide evidence-based treatments.

According to Sammarco et al., the most common findings resulting in tarsal tunnel syndrome were scars, vascular leashes and varicosities, therefore, our described technique of minimizing surgical damages might be important [45]. 92 patients with a carpal tunnel syndrome operated by an ultra-minimally invasive procedure got the same neurologic recovery with earlier functional return and less postoperative morbidity as mini-open carpal tunnel release [40].

In relation to the carpal tunnel and ultrasound-guided surgery, there are promising data that shed new lights over the sono-surgical releases of the nerves such as UGAFDS. The tarsal tunnel´s topography is different compared to the carpal tunnel; the tarsal tunnel is anatomically more complicated, it contains neurovascular bundles with difficult branching patterns, more osteo-fibrous tubes; it is influenced by weight bearing, biomechanics and footwear.

During the release of the flexor retinaculum with our UGAFDS approach, one has to keep in mind that, especially for not trained US-users, tools such as power doppler are crucial to visualize perforating veins to avoid accidental cuts [29].

However, we advocate that biomechanics of the foot and lower extremities play an important role in the tarsal tunnel syndrome. Precisely, dynamic pressure measurements in closed kinetic chain should be investigated, to get a better comprehension of the pathophysiological mechanisms involved in this issue.

## Conclusion

The results of our anatomic study indicate that our new minimally invasive, ultrasound-guided ankle and foot decompression surgery technique (UGAFDS) for the release of the flexor retinaculum (laciniate ligament) at the proximal tarsal tunnel is an effective, safe, quick and less expensive technique to treat selected patients with proximal tarsal tunnel syndrome. This technique reduces damage of the soft tissue and might therefore avoid scars and tissue fibrosis and minimizes the postoperative complications as seen in open surgery.

## References

[1] Alshami AM, Cairns CW, Wylie BK, Souvlis T, Coppieters MW (2009). Reliability and size of the measurement error when determining the cross-sectional area of the tibial nerve at the tarsal tunnel with ultrasonography. Ultrasound Med Biol.

[2] Alshami AM, Souvlis T, Coppieters MW (2008). A review of plantar heel pain of neural origin: differential diagnosis and management. Man Ther.

[3] Antoniadis G, Scheglmann K (2008). Posterior tarsal tunnel syndrome: diagnosis and treatment. Dtsch Arztebl Int.

[4] Barrett SJ, O’Malley R (1999). Plantar fasciitis and other causes of heel pain. Am Fam Physician.

[5] Beard NM, Gousse RP (2018). Current Ultrasound Application in the Foot and Ankle. Orthop Clin N Am.

[6] Butler DS (2000). The sensitive nervous system.

[7] Cimino WR (1990). Tarsal tunnel syndrome: review of the literature. Foot Ankle.

[8] Daniels TR, Lau JT, Hearn TC (1998). The effects of foot position and load on tibial nerve tension. Foot Ankle Int.

[9] Day FN, Naples JJ (1996). Endoscopic tarsal tunnel release: update 96. J Foot Ankle Surg.

[10] Delfaut EM, Demondion X, Bieganski A, Thiron MC, Mestdagh H, Cotten A (2003). Imaging of foot and ankle nerve entrapment syndromes: from well-demonstrated to unfamiliar sites. Radiographics.

[11] Dellon A (1984). Tinel or not tinel. J Hand Surg.

[12] Dellon AL, Mackinnon SE (1984). Tibial nerve branching in the tarsal tunnel. Arch Neurol.

[13] Erickson SJ, Quinn SF, Kneeland JB, Smith JW, Johnson JE, Carrera GF, Shereff MJ, Hyde JS, Jesmanowicz A (1990). MR imaging of the tarsal tunnel and related spaces: normal and abnormal findings with anatomic correlation. AJR Am J Roentgenol.

[14] Franson J, Baravarian B (2006). Tarsal tunnel syndrome: a compression neuropathy involving four distinct tunnels. Clin Podiatr Med Surg.

[15] García EN (2017) Principios de la cirugía mínimamente invasiva. Cirugía mínimamente invasiva del pie 23

[16] Gould JS (2011). Tarsal tunnel syndrome. Foot Ankle Clin.

[17] Gupta R, Rummler LS, Palispis W, Truong L, Chao T, Rowshan K, Mozaffar T, Steward O (2006). Local down-regulation of myelin-associated glycoprotein permits axonal sprouting with chronic nerve compression injury. Exp Neurol.

[18] Hendrix CL, Jolly GP, Garbalosa JC, Blume P, DosRemedios E (1998). Entrapment neuropathy: the etiology of intractable chronic heel pain syndrome. J Foot Ankle Surg.

[19] Iborra A, Villanueva M, Barrett SL, Rodriguez-Collazo E, Sanz P (2018). Anatomic delineation of tarsal tunnel innervation via ultrasonography. J Ultrasound Med.

[20] Jolly GP, Zgonis T, Hendrix CL (2005). Neurogenic heel pain. Clin Podiatr Med Surg.

[21] Joshi S, Joshi S, Athavale S (2006). Anatomy of tarsal tunnel and its applied significance. J Anat Soc India.

[22] Keck C (1962). The tarsal-tunnel syndrome. J Bone Joint Surg.

[23] Kelikian AS, Sarrafian SK (2011). Sarrafian’s anatomy of the foot and ankle: descriptive, topographic, functional.

[24] Kim E, Childers MK (2010). Tarsal tunnel syndrome associated with a pulsating artery: effectiveness of high-resolution ultrasound in diagnosing tarsal tunnel syndrome. J Am Podiatr Med Assoc.

[25] Kinoshita M, Okuda R, Morikawa J, Jotoku T, Abe M (2001). The dorsiflexion-eversion test for diagnosis of tarsal tunnel syndrome. J Bone Joint Surg Am.

[26] Konschake M, Brenner E (2014). “Mors auxilium vitae”—Causes of death of body donors in an Austrian anatomical department. Ann Anat.

[27] Kopell HP, Thompson WA (1960). Peripheral entrapment neuropathies of the lower extremity. N Engl J Med.

[28] Krishnan KG, Pinzer T, Schackert G (2006). A novel endoscopic technique in treating single nerve entrapment syndromes with special attention to ulnar nerve transposition and tarsal tunnel release: clinical application. Neurosurgery.

[29] Kuster G (1968). Anatom of the veins of the foot. Surg Gynec Obstet.

[30] Lam SJ (1967). Tarsal tunnel syndrome. J Bone Joint Surg Br.

[31] Lau JT, Daniels TR (1999). Tarsal tunnel syndrome: a review of the literature. Foot Ankle Int.

[32] Lee D, Dauphinee DM (2005). Morphological and functional changes in the diabetic peripheral nerve—using diagnostic ultrasound and neurosensory testing to select candidates for nerve decompression. J Am Podiatr Med Assoc.

[33] Mann RA (1976). Orthopedics: tarsal tunnel syndrome. West J Med.

[34] McHanwell S, Brenner E, Chirculescu A, Drukker J, van Mameren H, Mazzotti G, Pais D, Paulsen F, Plaisant O, Caillaud M (2018). The legal and ethical framework governing Body Donation in Europe-A review of current practice and recommendations for good practice. Eur J Anat.

[35] Mook WR, Gay T, Parekh SG (2013). Extensile decompression of the proximal and distal tarsal tunnel combined with partial plantar fascia release in the treatment of chronic plantar heel pain. Foot Ankle Spec.

[36] Nagaoka M, Matsuzaki H (2005). Ultrasonography in tarsal tunnel syndrome. J Ultrasound Med.

[37] Padua L, Aprile I, Pazzaglia C, Frasca G, Caliandro P, Tonali P, Martinoli C (2007). Contribution of ultrasound in a neurophysiological lab in diagnosing nerve impairment: a one-year systematic assessment. Clin Neurophysiol.

[38] Patel AT, Gaines K, Malamut R, Park TA, Toro DR, Holland N, Electrodiagnostic M, American Association of N (2005). Usefulness of electrodiagnostic techniques in the evaluation of suspected tarsal tunnel syndrome: an evidence-based review. Muscle Nerve.

[39] Riazi S, Bril V, Perkins BA, Abbas S, Chan VW, Ngo M, Lovblom LE, El-Beheiry H, Brull R (2012). Can ultrasound of the tibial nerve detect diabetic peripheral neuropathy?. A cross-sectional study. Diabetes Care.

[40] Rojo-Manaute JM, Capa-Grasa A, Chana-Rodriguez F, Perez-Mananes R, Rodriguez-Maruri G, Sanz-Ruiz P, Munoz-Ledesma J, Aburto-Bernardo M, Esparragoza-Cabrera L, Cerro-Gutierrez MD, Vaquero-Martin J (2016). Ultra-minimally invasive ultrasound-guided carpal tunnel release: a randomized clinical trial. J Ultrasound Med.

[41] Rojo-Manaute JM, Capa-Grasa A, Rodriguez-Maruri GE, Moran LM, Martinez MV, Martin JV (2013). Ultra-minimally invasive sonographically guided carpal tunnel release: anatomic study of a new technique. J Ultrasound Med.

[42] Rojo-Manaute JM, Rodriguez-Maruri G, Capa-Grasa A, Chana-Rodriguez F, Soto Mdel V, Martin JV (2012). Sonographically guided intrasheath percutaneous release of the first annular pulley for trigger digits, part 1: clinical efficacy and safety. J Ultrasound Med.

[43] Rose JD, Malay DS, Sorrento DL (2003). Neurosensory testing of the medial calcaneal and medial plantar nerves in patients with plantar heel pain. J Foot Ankle Surg.

[44] Rosson GD, Larson AR, Williams EH, Dellon AL (2009). Tibial nerve decompression in patients with tarsal tunnel syndrome: pressures in the tarsal, medial plantar, and lateral plantar tunnels. Plast Reconstr Surg.

[45] Sammarco GJ, Chang L (2003). Outcome of surgical treatment of tarsal tunnel syndrome. Foot Ankle Int.

[46] Schon LC, Glennon TP, Baxter DE (1993). Heel pain syndrome: electrodiagnostic support for nerve entrapment. Foot Ankle.

[47] Sessions J, Nickerson DS (2014). Biologic basis of nerve decompression surgery for focal entrapments in diabetic peripheral neuropathy. J Diabetes Sci Technol.

[48] Shacklock M (2005). Clinical neurodynamics: a new system of musculoskeletal treatment.

[49] Sofka CM, Collins AJ, Adler RS (2001). Use of ultrasonographic guidance in interventional musculoskeletal procedures - A review from a single institution. J Ultrasound Med.

[50] Takakura Y, Kitada C, Sugimoto K, Tanaka Y, Tamai S (1991). Tarsal tunnel syndrome. Causes and results of operative treatment. J Bone Joint Surg Br.

[51] Tassler PL, Dellon AL (1996). Pressure perception in the normal lower extremity and in the tarsal tunnel syndrome. Muscle Nerve.

[52] Tawfik EA, El Zohiery AK, Abouelela AA (2016). Proposed sonographic criteria for the diagnosis of idiopathic tarsal tunnel syndrome. Arch Phys Med Rehabil.

[53] Trepman E, Kadel NJ, Chisholm K, Razzano L (1999). Effect of foot and ankle position on tarsal tunnel compartment pressure. Foot Ankle Int.

[54] van Maurik JFM, Schouten ME, ten Katen I, van Hal M, Peters EJ, Kon M (2014). Ultrasound findings after surgical decompression of the tarsal tunnel in patients with painful diabetic polyneuropathy: a prospective randomized study. Diabetes Care.

[55] Watson TS, Anderson RB, Davis WH, Kiebzak GM (2002). Distal tarsal tunnel release with partial plantar fasciotomy for chronic heel pain: an outcome analysis. Foot Ankle Int.

[56] Wieman TJ, Patel VG (1995). Treatment of hyperesthetic neuropathic pain in diabetics. Decompression of the tarsal tunnel. Ann Surg.

